# Hospital Relief in Holland

**Published:** 1910-12-31

**Authors:** 


### HOSPITAL RELIEF IN HOLLAND.

### THE DUTCH SYSTEM, AND HOW IT WORKS.

We have recently heard a good deal in favour of the German system of hospital relief. At the conference of the British Hospitals Association held at Glasgow Mr. Thies, among others, pointed out the excellences of this system, and suggested that one way out of the difficulties which confront us in dealing with the vexed problem of hospital abuse would be to adopt this system, or such modification of it as lends itself to our somewhat different social conditions. Everyone who has studied hospital questions in Germany must have been struck with the solidarity that prevails in the institutional world in that country. It is interesting to discuss the causes of such solidarity, and to debate the point in how far the latter rests upon the active support which the State and the various municipalities give to the charitable institutions. We have lately published articles showing how the system of compulsory invalidity and sickness insurance has reacted upon German hospitals, and in discussing the whole question of medical relief in Germany the existence of that system of compulsory thrift must never be lost sight of. It is therefore interesting, for purposes of comparison, to turn to a country in which compulsory insurance does not as yet exist in the same plenary fashion as it does in Germany, and it seems to us that a brief summary of the conditions prevailing in the best hospitals of Holland may be of great value to institutional workers who are actively concerning themselves in the prevention of hospital abuse.

We have been in correspondence with Dr. van Eysselsteyn, the director of one of the largest and best known of the magnificent institutions which have lately been established in Holland, and he has been good enough to favour us with his views on the working of the system which obtains in the Netherlands. In reviewing the Dutch system it is worth bearing in mind that Holland is, in comparison with Germany, a far less "State-ridden" country. Leaving aside wholly the question whether State interference in social matters is reprehensible or the reverse, it may be suggested that such interference is bound, in the long run, to influence the economic relations between hospitals and their population. Mr. Burritt's able exposition of the subject, published in THE HOSPITAL of November 21, shows the truth of this suggestion, and the development of hospitals in Germany, as described by Grotjahn, offers yet more convincing proof of its reality.

The Dutch system is closely modelled on that of the Germans, but it differs from the latter in several important particulars. The rules of the Groningen General Hospital, which may be taken as a type of the regulations which control the whole system, are very definite. As given in the official journal of the institute they are as follows:—

A deposit of 100 florins in the case of first- and second-class patients, or of 45 florins in the case of third-class patients, is required in every case on the admission of patients, provided that the patient is unable to produce a certificate showing clearly that some corporation, society, or association is willing to make itself wholly responsible for the cost of such in-patient's treatment while in hospital, or a certificate from a member of the staff of the hospital stating that in his opinion such a patient is a fit subject for clinical demonstration, in which case the cost of the treatment is debited to the State.

The deposit is placed to the patient's account for expenses while under treatment. A written receipt is in all cases given.

Patients on admission are placed in the division in which they are to be treated. The choice of division rests with the director, who acts on the advice of the staff.

Soldiers below the rank of non-commissioned officers are placed in the director's own division, provided they are not suffering from contagious disease.

Officers are ranked as patients of the first class, and subalterns as patients of the second class in their respective divisions.

Non-commissioned officers and cadets are treated apart from the other military patients where such separation is possible or advisable.

Attendants responsible for the transport of patients to the hospital are provided with a blank form. [This form is simply an undertaking on the part of the patient that he or she will conform to the rules of the establishment and guarantees to pay the costs of the treatment.] The form must be signed by the patient or those responsible for him in the presence of two witnesses, and is to be lodged in the director's office on the patient's admission into hospital.

Three days before the deposit expires, in the event of the patient being still under treatment, the patients, or those responsible for them, are to be notified and called upon to provide further moneys or to authorise further expenditure on their behalf.

Patients for whose expenses the municipality, church, or benevolent funds are responsible, and who reside beyond the limits of the province of Groningen, are only admitted provided there are sufficient beds available for the purpose.

Patients for whose expenses charitable or other funds are responsible can only be admitted after such funds have undertaken the full responsibility of defraying all necessary expenses.

Patients admitted at the request of private persons who have volunteered to defray their expenses are treated as special patients at a payment of such charge as the Mayor and Councillors may from time to time determine. First- and second-class patients are not bound to submit themselves to members of the staff for teaching purposes. Those of the first class are allowed, with the consent of members of the staff, to call in their private medical attendants if they choose to do so.

Out-patients are only treated gratuitously if they are in a position to prove that they are unable to pay for their treatment. The onus of proof rests on them, and they must provide themselves with a proper certificate signed by a municipal or ecclesiastical authority, stating that they are not in a position to pay the fees.

Members of the hospital staff have the privilege of disposing of a certain number of free days, reckoned at the rate of not more than 4,000 during the year, for the benefit of such patients as they may consider fit subjects for purposes of clinical demonstration. Such patients shall only be admitted provided there are beds available for them. The cost of their treatment, reckoned at the rate charged for municipal or benevolent societies' patients, shall be borne by the State.

The following are copies of the notices forwarded to every patient or his medical attendant or friends before admission:—

Groningen.

Herewith I beg to inform you that the patient on whose behalf you requested a bed in the —— department of the hospital, can be admitted on condition that he (or she) is provided with a certificate to the effect that the municipality is prepared to pay the costs of treatment unless the patient enters as a private patient, in which case he (or she) must be prepared to deposit a sum of 45 florins (approximately £3 15s.), which suffices for thirty days' treatment at the rate of 1.50 florins per day. In case the patient has to be transported from the train or boat to the hospital, I shall be glad if you will inform me of this fact as early as possible in order that the necessary arrangements may be made.

The Medical Superintendent, Dr. G. van Eysselsteyn.

Groningen.

Private patients, from any locality, pay at the following rates: First class, 10s. per day (separate room); second class, 5s. per day (two in one room); third class, 2s. 6d. per day (in the wards).

For third-class patients this sum includes everything.

In the case of patients of the first and second class, medicines, wine, bandages, laundry, and private consultations or operations are regarded as extras.

All payments for patients belonging to any of these classes must be made on the day of admission, and a sum sufficient to pay for the expenses of thirty days' hospital treatment must be deposited.

Benevolent societies pay at the rate of 2s. per day per patient, subject, provisionally, to whatever further charge may be made at the end of a year in accordance with the expenses of treatment.

The Director of the Hospital, Dr. G. van Eysselsteyn.

Commenting on these regulations, Dr. van Eysselsteyn writes: "You will observe that circumstances here appear to be wholly different from what they are in England. Here we admit no one unless he deposits the cost of thirty days' hospital treatment previous to his admission, or unless he brings with him a guarantee from some fund that such expenses will be paid for him. The sole exception to this is in the case of patients who are thought suitable subjects for teaching purposes. These are admitted on the recommendation of responsible members of the staff who have been given a maximum of 4,000 free days of treatment for the benefit of such patients. Even in their case we must be satisfied that the patient himself is not in a position to pay the expenses of treatment. . . . In the wards we have paying patients as well as poor patients—*i.e.* those whose expenses are paid by the State or by the funds. I have never experienced that this system gives rise to any complaints. The better class patients—schoolmasters, tradesmen, etc.—usually contrive to keep to themselves and appropriate one end of the day-room, while the poorer patients seem to shake down as easily at the other end. As a matter of fact, the arrangement works admirably, and a proof of its popularity is found in the fact that our number of third-class paying patients is every year increasing."

In many ways this system of admitting paying patients to the general wards is on a similar footing with the system that prevails in Germany, although in the latter country an attempt is usually made to sort out the paying from the poor patients, and to provide separate wards for them both. The advantages of the Dutch system appear to be great, in view of the fact that it economises expense, admits of a smaller *personnel*, and—a point of some practical importance—to some extent robs the poor ward of its pauper character. Specially worthy of attention is the strict regulation of the out-patient department. It may be imagined that the proviso that non-paying out-patients must produce signed certificates of poverty opens the door to abuse in this department; but, as a matter of fact, the system appears to answer admirably. It obviates the necessity of employing a hospital almoner, since it throws on the patient, and not on the hospital authorities, the task of proving that the former is a legitimate object for charitable treatment.

We have devoted some space to the rules obtaining at Groningen, since the Dutch system is not so well understood in this country as it ought to be. Of late years hospital politics have been extensively discussed in Holland. New hospitals have sprung up; modern up-to-date methods have been instituted; and no one who wishes to study the whole question of charitable relief can afford to disregard the striking developments that have taken place in that country. In many ways the problem of pauperism in Holland is supremely interesting. It has aspects which are quite different from those of the problem as presented to us in England, and it is remarkable that hospital abuse, in the sense that we understand it here, is so little prevalent there. Perhaps the admirable system, of which the Groningen Hospital presents the best example, is one of the causes of this relative absence of abuse. At any rate, the system is worth studying, and to those who wish to know more about it in detail we recommend Dr. van Eysselsteyn's account of the work done at his institution in the yearly reports issued by the Groningen General Hospital.

